# Can ageing be beneficial?

**DOI:** 10.18632/aging.101313

**Published:** 2017-10-26

**Authors:** Stephen Frenk, Jonathan Houseley

**Affiliations:** Epigenetics Programme, Babraham Institute, Cambridge, UK

**Keywords:** aging, specialist, generalist, evolution of aging

Few questions pertain better to the modern human condition than ‘why do we age?’. The idea that aging results from an inevitable accumulation of wear-and-tear through life seems entirely natural and consistent with the world around us, and would simply explain the progressive loss of fitness with age. Scientific theories of aging have generally been consistent with this idea, formalised by Peter Medawar in the 1950s [1], and have coalesced on the view that aging is the inevitable result of compromises between processes under natural selection: either that division of limited resources for repair tends to favour germline maintenance at the expense of somatic tissue (Disposable Soma [2]); or, more generally, that fecundity is under stronger selection than longevity resulting in the emergence of traits that are advantageous for fecundity even if detrimental to longevity (Antagonistic Pleiotropy [3]). Conversely, an undercurrent of opinion has long suggested that aging is a defined process with a positive outcome that evolved under natural selection (Programmed Aging reviewed in [4]); such arguments have remained highly controversial however, principally because no fitness advantage of aging has been described that could sufficiently outweigh the obvious detriments.

Fitness is, in itself, a complex property encompassing both the intrinsic health of an organism and its fit to the current environment, such that an organism with high fitness in one environment may fare poorly in another irrespective of its intrinsic health. In general, organisms that are highly optimised towards growth in one environment are classed as specialists, while those which perform similarly across different environments are generalists. In the context of aging, this made us question the extent to which age-linked loss of fitness actually stems from a loss of intrinsic health, and how much could be attributed to a loss of specialisation to the current environment. The question is important as a high contribution from loss of specialisation would mean that aging cells may perform better when subject to conditions that depart from the environment for which the organism is specialised.

**Figure 1:**
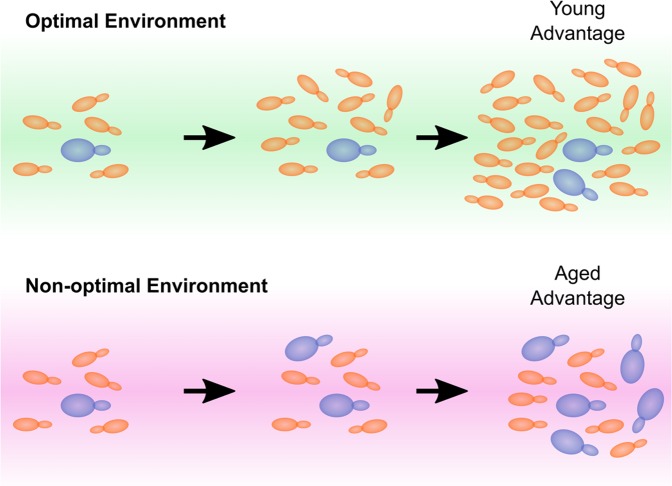
Aging cells thrive in non-optimal environments Whereas the aging cells in a population are at a competitive disadvantage during growth on media for which the organism is specialised, we observe that aging yeast cells have a competitive advantage in non-optimal conditions. This provides an unexpected selective advantage for aging cells when the population is exposed to non-optimal or fluctuating environments.

Recently, we addressed this experimentally in budding yeast, which is highly specialised for growth on glucose as a carbon source [5]. We competed young yeast cells against cells aged to their median lifespan. One might expect such cells to be extremely frail and indeed they reproduced poorly in glucose, the preferred carbon source, compared to their young counterparts. However, the aged cells showed dramatically higher fitness on other carbon sources such as galactose or acetate and robustly outcompeted the young cells (Figure 1). Remarkably, aging in galactose was accompanied by improvements in cell cycle time and homogeneity, suggesting that fitness actually increases with age on this carbon source. In other words, aging does not necessarily constitute a loss of intrinsic health in budding yeast but rather a transition from specialist to generalist.

These results are consistent with the idea that much of the observed fitness decline in aging yeast actually represents a loss of specialisation. This is very surpris-ing as aged yeast show a spectrum of molecular changes generally associated with aging pathology including genome instability, protein aggregation and chromatin de-repression, along with transcriptomic changes coherent with those observed during aging in higher eukaryotes (reviewed in [6]). Each of these changes would, in itself, be considered a pathology with a negative impact on intrinsic health, raising the interesting question of whether some of these changes offer an advantage to a generalist, or whether they are just by-stander events whose detrimental impact is insufficient to offset whatever other process drives the increased fitness of the aged cells in some environments.

So is aging truly the result of inevitable compromises? Likely yes: specialism versus generalism is simply another trade-off associated with age. However, this compromise is not one that is generally expected; our data shows that the fitness outcome of apparently pathological molecular changes depends on diet, raising questions as to whether select nutritional changes could suppress the impact of aging. Although our research was performed in yeast, a recent study has also questioned the nature of age-linked compromises in fruit flies; lifespan extension through dietary restriction was previously believed to be inseparably linked with reduced fecundity but it has now been shown that careful diet optimisation yields equivalent lifespan extension without impacting fecundity [7].

Overall, our findings demonstrate an unexpected potential for aging to be under positive selection, at least in unicellular eukaryotes. Although young budding yeast cells may be fitter under optimal conditions, a positive selection for aging would apply when cells are exposed to environments that are non-optimal or fluctuating. The former would, of course, also provide a selective pressure for a change in specialisation, but the latter would likely favour the sort of population heterogeneity in specialisation we observe to be generated by the aging process.
